# Reversing wetland death from 35,000 cuts: Opportunities to restore Louisiana’s dredged canals

**DOI:** 10.1371/journal.pone.0207717

**Published:** 2018-12-14

**Authors:** R. Eugene Turner, Giovanna McClenachan

**Affiliations:** 1 Louisiana State University, Department of Oceanography and Coastal Sciences, Baton Rouge, Louisiana, United States of America; 2 University of Central Florida, Orlando, Florida, United States of America; Texas A&M University, UNITED STATES

## Abstract

We determined the number of permits for oil and gas activities in 14 coastal Louisiana parishes from 1900 to 2017, compared them to land loss on this coast, and estimated their restoration potential. A total of 76,247 oil and gas recovery wells were permitted, of which 35,163 (46%) were on land (as of 2010) and 27,483 of which are officially abandoned. There is a direct spatial and temporal relationship between the number of these permits and land loss, attributable to the above and belowground changes in hydrology resulting from the dredged material levees placed parallel to the canal (spoil banks). These hydrologic modifications cause various direct and indirect compromises to plants and soils resulting in wetland collapse. Although oil and gas recovery beneath southern Louisiana wetlands has dramatically declined since its peak in the early 1960s, it has left behind spoil banks with a total length sufficient to cross coastal Louisiana 79 times from east to west. Dragging down the remaining material in the spoil bank back into the canal is a successful restoration technique that is rarely applied in Louisiana, but could be a dramatically cost-effective and proven long-term strategy if political will prevails. The absence of a State or Federal backfilling program is a huge missed opportunity to: 1) conduct cost-effective restoration at a relatively low cost, and, 2) conduct systematic restoration monitoring and hypothesis testing that advances knowledge and improves the efficacy of future attempts. The price of backfilling all canals is about $335 million dollars, or 0.67% of the State’s Master Plan for restoration and a pittance of the economic value gained from extracting the oil and gas beneath over the last 100 years.

## Introduction

**Fig 1:**
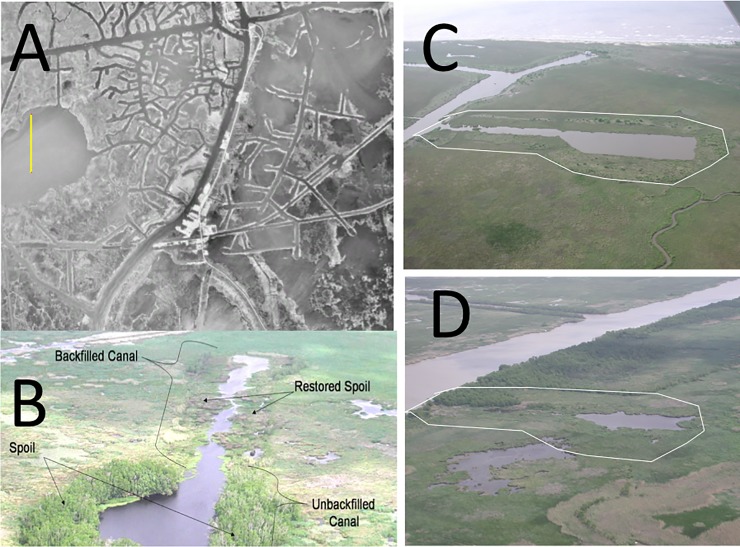
Photographic montage of canals and spoil banks. A. The Leeville, La, oil field with a mosaic of canals, wetlands and open water in 1989. The yellow vertical bar over Catfish Lake is 2 km. B. A canal that is not backfilled at the keyhole in the bottom of the picture, but is backfilled towards its entrance. C. A canal before being backfilled. D. The canal in C, after it was backfilled. The keyhole is the enlarged terminal end on the right of the dredged channel. A white outline of the shape of the spoil bank area is shown. The average width of the canals is about 33 m. Photo credits: A is a Landsat image sourced from the U.S. Geological Survey EarthExplorer (http://earthexplorer.usgs.gov/) and is freely available public domain data [4]. B-D are by the co-authors.

Dredged materials from the digging of canals in wetlands are often disposed of by creating continuous levees (also know as ‘spoil banks’). These dredged materials are aligned parallel to the canal on both sides. The levee height may be multiple times the tidal range and the deposited spoil material compresses the soil beneath with the effect of inhibiting both overland and belowground water flows [1,2]. Upland habitat is created that may be colonized by trees and shrubs covering an area as wide as the newly created canal (Fig 1). This industry practice creates linear waterways on a grand scale in order to begin recovering the oil and gas thousands of meters below. It is done worldwide [3].

The direct impacts of building canals and spoil banks peaked from 1955/56 to 1978, when about 7% of the coastal wetlands area in 1978 was open water [5]. There were additional wetland losses from the indirect effects of canals and spoil banks. The mechanisms identified to explain these indirect causes involves the consequences from changing wetland hydrology. This hydrologic damming above- and below-ground creates waterlogged soils that may lead to toxic sulfide accumulations [6] and reduces the accumulation of soil organic matter which dominates vertical accretion rates; the same damming effect causes longer drying cycles which leads to soil oxidation [2,7,8]. A site-specific example is on the south side of Jug Lake, west of Houma, LA. There open water increased from about 15% to 85% within 2 years after dredging [9]. Ponds formed near canals, but not a few kms away, and particularly where two or more spoil banks intersect [10]. Other examples are the wetland-to-open water conversions for 27 salt marshes (total = 6387 ha) in the Barataria, Breton Sound and Terrebonne estuaries from 1955 to 1990; open water area increased when dredging increased, and stabilized or slightly declined when dredging ceased [11].

The apparent temporal and spatial synchrony of canal density growth and wetland losses were not apparent until there was significant geospatial documentation of land. Coastal land area in Louisiana could not be measured until the 1930s because: 1) photographic records did not exist, 2) coastal maps in the 1890s recorded only stylized representation of ponds, small streams, and land, or 3) the surveyed land was too far from secure reference points. The loss rates from the 1930s to 1990 were measured using 5 sets of photographic records by Britsch and Dunbar [12] in a consistent manner. Couvillion et al. [13] used these detailed photographic interpretations and a combination of others, including satellite imagery, whose final pixel size was 30 m. The combined data sets describe a rise and fall in land loss whose rates are statistically indistinguishable from zero—they range from 410 to -526 km^2^ y^-1^ in 20 intervals from the early 1930s to 2016, with a slight reversal (gain) from 2010 to 2016.

The State of Louisiana proposes to restore some of these wetlands through an ambitious restoration program called the Master Plan [14]. The Master Plan is updated every five years and presently totals $50 billion, with $25 billion for restoration and $25 billion for flood protection infrastructure. The majority of the money allocated to restoration is split between 3 categories: marsh creation ($17.8 billion), sediment diversions ($5.1 billion), and barrier island restoration ($1.5 billion). There is no identified strategy within the Master Plan to restore canals by filling in the canal with the spoil bank material. This process is called ‘backfilling’ and is intended to restore marsh on the spoil bank and perhaps in the marsh. The progress of backfilling restoration success has been followed several times over the last 35 years at 33 locations and with favorable and predictable outcomes, and with virtually no negative consequences [15,16,17]. The backfilling benefits increase over time, although complete restoration may take longer than twenty years. Backfilling, it seems, could be quickly implemented coastwide and directly addresses a cause-and-effect driver of coastal wetland loss.

Backfilling, however, has been limited to these 33 canals and a few others [18,19,20]. Some obstacles to doing more are political [21], the sparse appreciation for the scale of the opportunities, and being unaware of the cause-and-effect relationships between dredging and land loss. Here we address two of these obstacles in an attempt to overcome the third. First, we count the number of canals permitted in south Louisiana from 1900 to 2017 and compare that number to land loss occurring in time and space. Second, we determine the potential number of permitted oil and gas wells that are classified as available for backfilling. Third, we re-visit the 33 canals backfilled in 1984 to determine whether the canal is plugged or not affects the width of the canal.

## Methods and materials

### Permit records

The permitting for oil and gas well drilling in Louisiana began in the early part of the last century and was accompanied by canal development where roads could not be developed–in wetlands [22]. The drilling permits issued by Louisiana from 1900 to 2017 are from the Department of Natural Resources, State of Louisiana website [http://sonris-www.dnr.state.la.us/gis/]. The individual permits include various data about the condition of the well, amount of mineral recovery, and details on drill depth, location and operator. We summarized the annual number of permits issued for 14 coastal parishes by year, and then overlaid their location on the coastal maps of Couvillion et al. [23] to determine where permits were still on land. These maps are the most recent ones available to us, and there was land-gain after 2010 [13]. The permits were further sorted into ‘abandoned’ or ‘plugged’ drilling wells. An abandoned drilling well “has no reported production, disposal, injection or other permitted activity for an extended period of time and the current operator of record has failed to maintain the oilfield site in accordance with state rules and regulations”. A plugged well is sealed “to stop open communication of formation fluids within a well” [Louisiana Department of Natural Resources guidelines, http://www.sonris.com/documents/FinalLouisianaDNRGlossaryofTerm.pdf]. In this way we built a temporal and spatial database of the number of drilled wells that might be backfilled if they were dredged in wetlands and listed as abandoned or plugged.

### Oil and gas production

The oil and gas production data for southern Louisiana are from Tables 1 and 9 of the Technology Assessment Division, Louisiana Energy Facts and Figures, Department of Natural Resources [http://www.dnr.louisiana.gov/index.cfm/page/208]. The southern region has produced more oil than the northern region, and the offshore region has never exceeded ten percent of the state’s production [24]. The amounts from 1945 to 2017 were normalized to a common scale of 0 to 1 by comparing the annual amount to the maximum.

### Land loss rates

We used the land loss rates measured for the whole coast (km^2^ y^-1^) by Britsch and Dunbar [12] who conducted a systematic analysis of aerial photography for four intervals: 1930s to 1956–1958, 1956–1958 to 1974, 1974 to 1983, and 1983 to 1990. These data are for 15 minute quadrangle maps and we excluded maps with less than 15% land, which include the highly mineral soils [25]. The data for land change from 1990 to 2010 are from Couvillion et al. [13]. The Couvillion et al. [13] data set uses a combination of satellite and aerial imagery whose pixel size was scaled up to 30 m. The Couvillion et al. [13] estimate of land loss was reduced in proportion to the area of land covered by Britsch and Dunbar’s analysis [12].

### Backfilling

Thirty-three backfilled sites were described by Turner et al. [16] and updated by Baustian and Turner [5]. These sites were revisited using aerial photographs in Google Earth for January 1998, March 2004, October 2010, and February 2017. Turner et al. [26] evaluated the precision of these images by making measurements of the distance between fixed points on structures for six different locations using seven to twelve different photographs for each site, with each representing a different photograph date. The standard error of the mean (SEM) for the widths of the fixed structures ranged from 0.09 to 0.33 m, with a coefficient of variation of 0.22 to 0.89%.

We used these Google Earth photographs to measure the width of a backfilled canal and a nearby reference canal. The keyhole is wider than the canal and is where the drilling hole is located and where dredging and maintenance vessels turn around (Fig 1B and 1C). A 200-m transect was placed within each canal using the measuring tool in Google Earth, from 10–20 m before the keyhole started at its terminal end and extending towards its entrance at the connecting waterway. The transect was segmented into 10 equally spaced sub-sections and the widths measured at the 11 perpendicular transect lines. Equivalent measurements were made in the nearest canal (minimum 200 m length) that was not backfilled and that appeared abandoned based on the absence of physical structures within the canal. There were 12 paired sites available, but only 11 longer than 200 m because one canal had inadequate backfilling that degraded the spoil bank surface too low for vegetation. Five of these 11 sites had plugs at their entrance in 1992 [16].

The measurements of widths were averaged for each canal by year and location, and the SEM were calculated. The % recovery was calculated as a percent of the width of the canal in the 1998 image. A two-tailed Student’s t-test was made to determine difference between the widths of the paired reference and backfilled sites; the level of statistical significance was *p* < 0.05. The average depth of the backfilled canal in 1992 [16] was compared to the canal width in 2017. The data set was divided into data sets that had canals that were plugged or not, as determined by an inspection of the aerial imagery.

### Canal length

A linear regression of the area of canal from Britsch and Dunbar [12] for each of the 5 dates was compared to the cumulative number of permits issued at that time. The resulting equation was used to extrapolate to the area of canals at the end of 2017 using the equation slope and then number of canals. The canal width of the 12 reference canals calculated above was divided into the canal area to estimate the length of canal and spoil banks in 2017.

## Results

### Permit records

**Fig 2:**
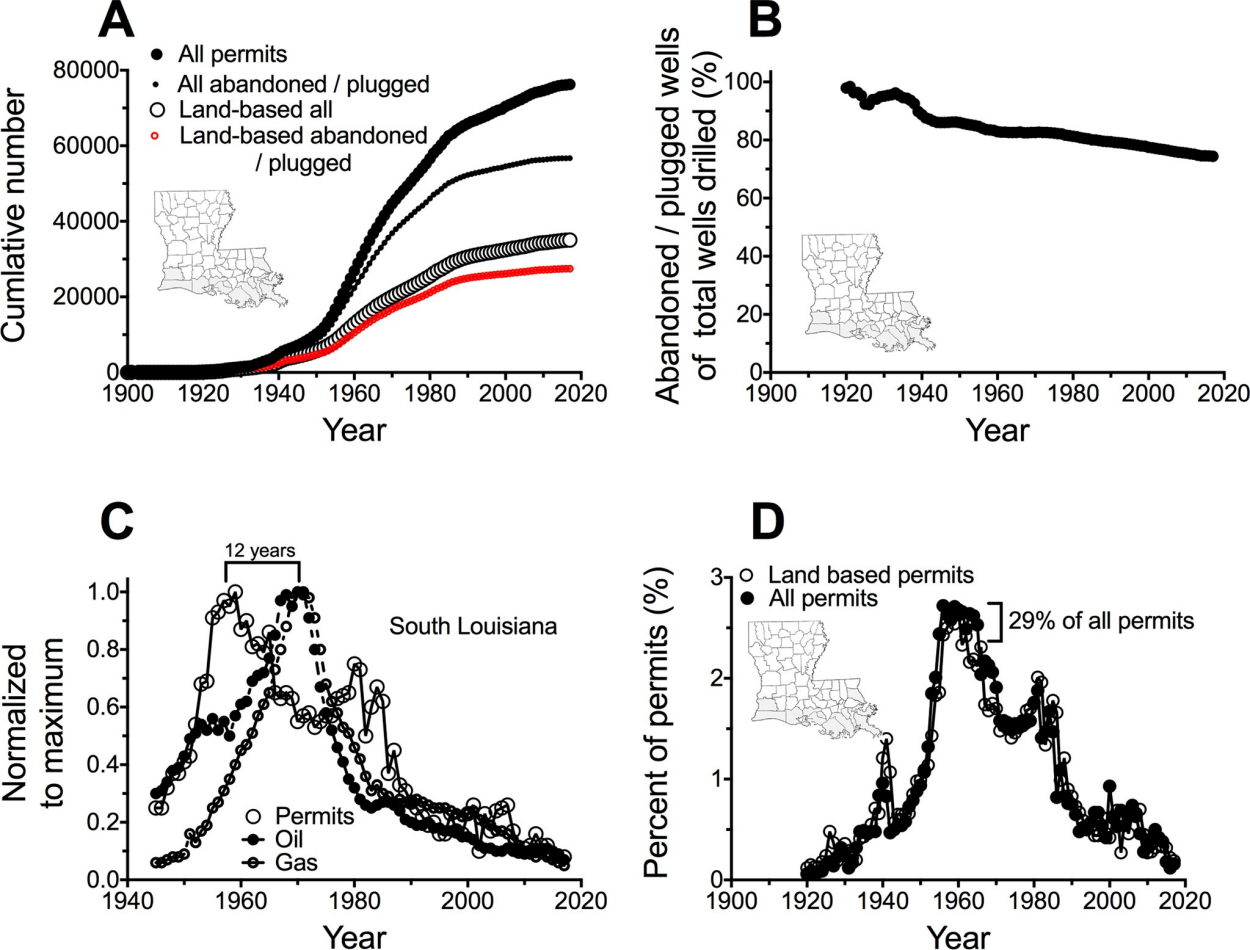
The rise in various kinds of oil and gas drilling permits in the 12 parishes of south Louisiana. A. The total permits, those labeled as abandoned or plugged in the permit files, the permits issued for land/wetland, and the land/wetland permits that are labeled as abandoned or plugged in the permit files. B. The cumulative percent of permits that are labeled abandoned and plugged by that year. C. The peaks in permit number and the oil and gas production (normalized). D. The percent of all permits issued each year and the % for land/wetland activity.

**Fig 3:**
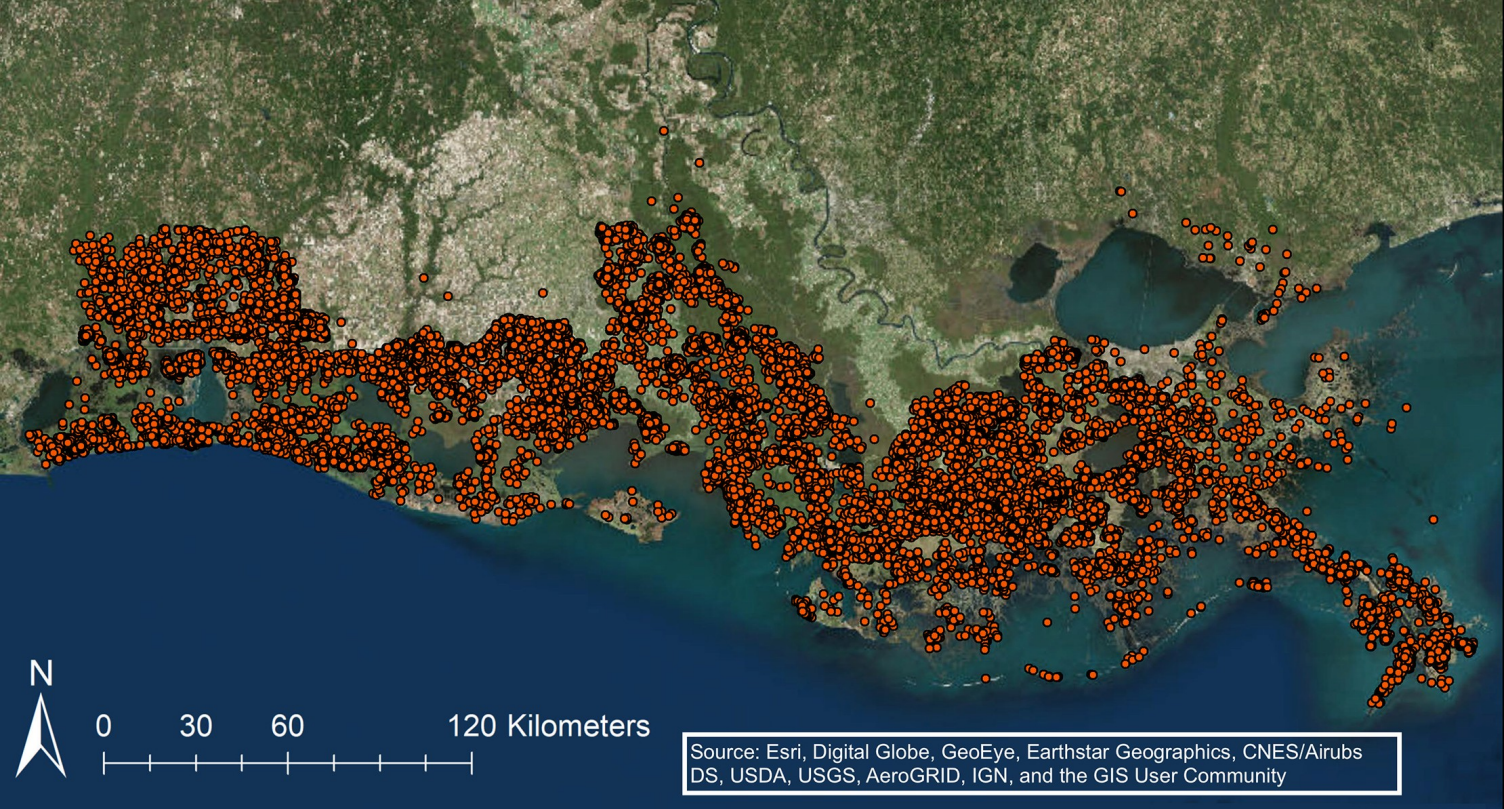
The distribution of oil and gas well permits issued between 1900 and 2017 that were ‘plugged’ or ‘abandoned’ in 2017 and on land in the 14 parishes.

A total of 76,247 wells were permitted in 14 parishes from 1 January, 1900 to 31 December, 2017, of which 35,163 (46%) were on land (Fig 2A). There were 56,719 wells labeled as plugged or abandoned (74%) as of 2017 (Fig 2A), of which 27,483 (36%) were on land. The total number of all permits drilled that were abandoned or plugged was above 95% in the first decades of the last century and has since declined to 81% of the total permits issued (Fig 2B). The total number of permits peaked in the late 1950s and early 1960s when about one third of all permits were issued (Fig 2C). The maximum in permits issued was in 1958, and the production of oil and gas in southern Louisiana peaked 12 years later (Fig 2D). These potential sites for backfilling canals are in all 14 parishes (Fig 3).

### Land loss rates

**Fig 4:**
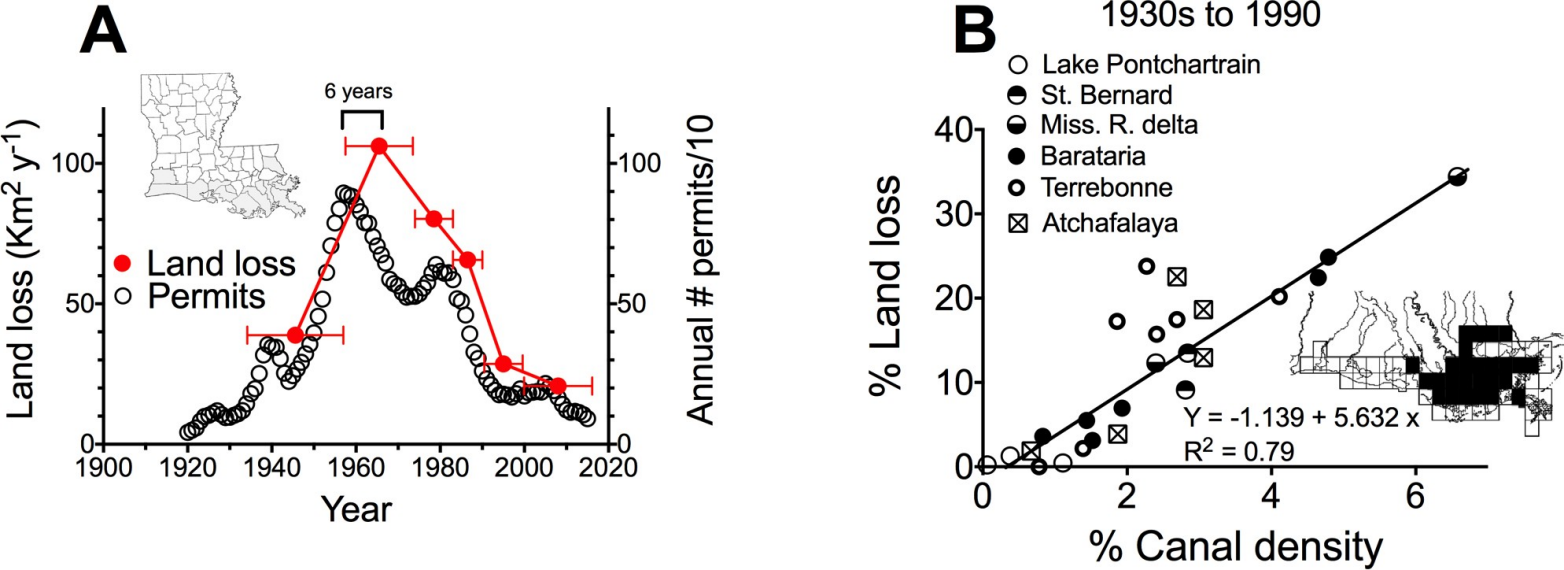
The number of oil and gas permits issued annually and the land loss rates. B. The land loss rate from the 1930s to 1990 and canal density in 15 minute quadrangle maps.

The temporal distribution of total wells permitted overlaps with the land loss rates for 5 intervals from 1930s to present (Fig 4A), and has a 6-year lag in the peak land loss rates. The spatial distribution of land loss from the 1930s to 1990 is that the land loss in 15 minute quadrangle maps is directly related to the % of canals in each map (Fig 4B). The slope of the intercept goes through zero and the slope of the linear regression line indicates that there is 4.6 times more land area lost indirectly than directly from the canal area created.

### Backfilling progress

**Fig 5:**
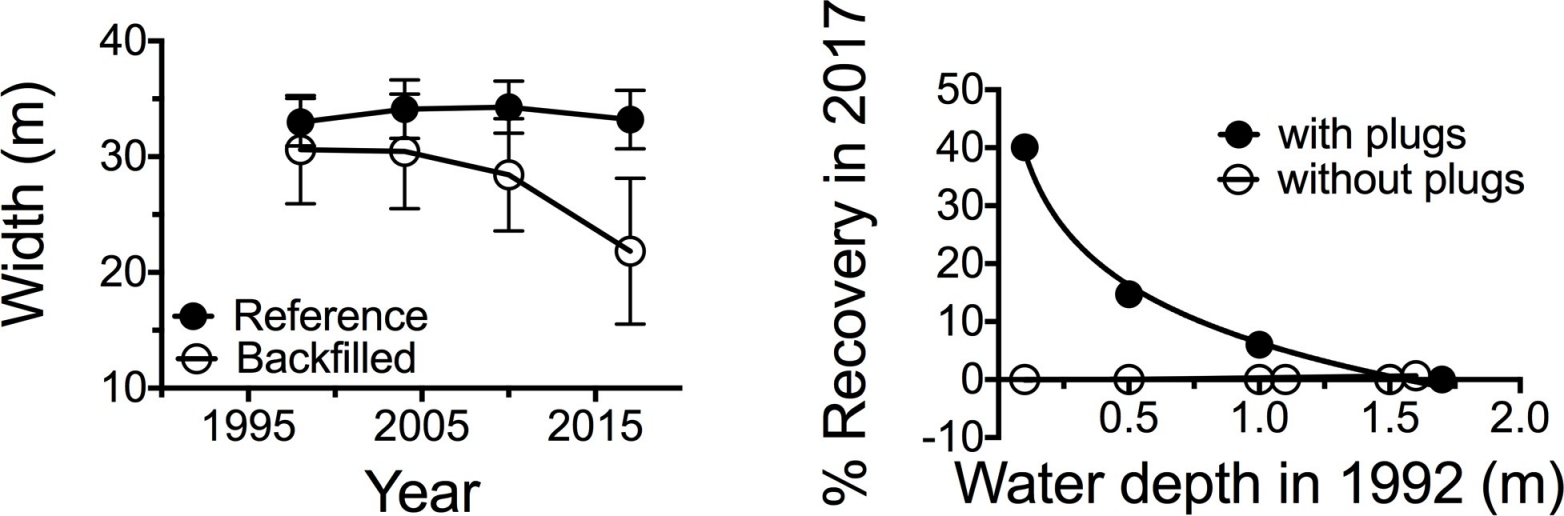
Changes in the width of backfilled and reference canals that were >200 m long in 1992. A: Widths of backfilled and reference canals in 1998, 2004, 2010 and 2017. B: Restoration from 1998 for backfilled canals versus the depth of the water in 1992 that were had plugs visible in the imagery. The means ± 1 SEM are shown.

The backfilled canal widths (doi: 10.7266/n7-7gs4-j475) were initially the same size as for the reference canals in 1998, 2004 and 2010, but were different by 2017 (*p* = 0.038) when the decline in average width of the backfilled canals was 69% of the average width in 1998 (Fig 5A). The restoration by 2017 increased as the water depth of the canal in 1992 declined. The shallower the canal depth, then the more vegetation in the plugged backfilled canals, but in not the unplugged canals (Fig 5B).

### Cumulative length of canals and spoil banks

**Fig 6:**
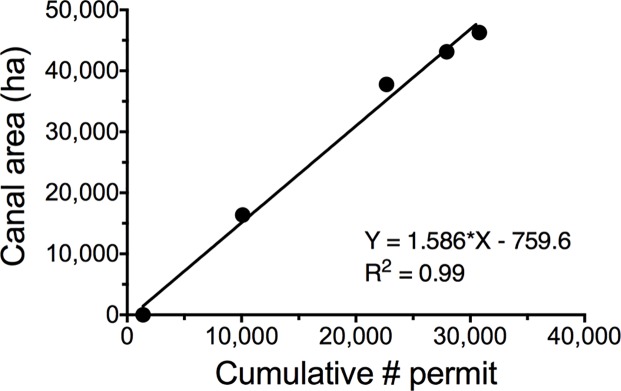
The canal area in coastal Louisiana for 5 dates from 1933 to 1990 and the cumulative permits issued by then. A simple linear regression of the data is shown.

There were 30,785 permits issued by the end of 1990, when there were about 46,725 ha of canals according to Britsch and Dunbar [12]. There were an average 1.586 ha of canal for every permit issued from the early 1930s to 1990 (Fig 6). A total of 35,163 permits were issued at the end of 2017, which implies that there were 55,783 ha of canals then. If we assume that the width of the 12 reference canals in 2017 (33.1 ± 0.23 m in 1994 and 33.3 ± 0.39 m in 2017) applies to all canals in 2017, then there are 16,853 km of canals and double that amount (33,705 km) of spoil banks.

## Discussion

The Louisiana coastal zone in 2017 had a cumulative total length of 33,705 km of spoil bank, which is more than 3/4ths of the circumference of the Earth. The total length of spoil banks in 2017 was long enough to cross the Louisiana coast east-to-west 79 times with a spoil bank height up to 3–10 times the natural tidal range. Clearly this is a significant factor influencing wetland health. In fact, the spatial and temporal distribution of permitting is not only coincidental with land loss, but the intercept of land loss and canal density in Fig 4B is zero, implying a dominant causal relationship.

Backfilling addresses the consequences to wetlands that the canal and spoil bank caused when built and afterwards. Backfilling restores wetland, prevents future wetland loss, and is highly cost effective… but may take decades to restore. The backfilling benefits increase over time, although complete restoration will take longer than twenty years. The State is focusing billions of dollars for unproven river diversion programs costing more per ha gained than backfilling, and it will decades to find out if diversions work [14]. Improving the completeness of spoil removal, coupled with appropriate site selection and plugs, will speed up the restoration process and enhance future backfilling projects. Backfilling can be quickly implemented across the coast and doing so directly addresses the main cause-and-effect driver of coastal wetland loss across the entire coast of Louisiana.

The 33 canals backfilled by 1984 were not backfilled in a systematic way or chosen to optimize restoration [15]. They were simply opportunities arising in the permitting office just before the program was stopped. Some are within an impoundment that has a larger influence on wetland hydrology than the smaller backfilled canal; others are in an area that was eroding before backfilling, and others are stubby canals without plugs, or are canals now used only for navigation, not mineral recovery. They were, in other words, a ‘hit-or-miss’ opportunity to do something rather than nothing [21].

### A proposal

Backfilling both prevents future land loss and restores land already lost, and there are 27,483 potential canals on land available for backfilling if will prevails and money becomes available. Many canals are supposed to be backfilled upon abandonment as part of the legal process, but are not. The absence of a State or Federal backfilling program is a huge missed opportunity to conduct cost-effective restoration that could be done at a relatively low cost [19]. The majority of coastal wetland area on this coast is privately owned, with the remainder in various public agencies including School Boards, non-Governmental Agencies, State and Federal Lands. It may take some organized and low-key persuasion, but canals could be backfilled within a program that was positively promulgated by State government. A bundling of many backfilled sites within one managerial effort would probably have economies of scale that doing one at a time do not; effective backfilling is partially dependent on operator skill [15], and a systematic monitoring. Including a hypothesis-testing program within an adaptive management framework [27] would advance restoration knowledge and future attempts.

The price of backfilling (without sediment added) was $9,266 ha^-1^ in 2005, and $12,224 ha^-1^ in 2018 when adjusted for inflation. The rough approximation of filling in all abandoned canals is, therefore, about $335 million dollars, or one-fifth of the cost of one river diversion. The total crude oil production since 1900 in the southern region was $613 billion at $60 per barrel, or 0.05% of the cost to restore all of the now abandoned or plugged canals on land in the same region. The State restoration plan is a minimum of $50 billion dollars over the next 50 years. We propose that 0.67% of that money be spent to reverse/restore the effects of the main cause of land lost across the whole coast.
